# Artificial Intelligence in Gynaecological Malignancies: Perspectives of a Clinical Oncologist

**DOI:** 10.7759/cureus.45660

**Published:** 2023-09-21

**Authors:** Himanshi Khattar, Ruchica Goel, Piyush Kumar

**Affiliations:** 1 Radiation Oncology, Shri Ram Murti Samarak Institute of Medical Sciences, Bareilly, IND; 2 Gynaecological Oncology/In Vitro Fertilization, Shri Ram Murti Samarak Institute of Medical Sciences, Bareilly, IND

**Keywords:** quality assurance, chemotherapy, radiotherapy, artificial intelligence, gynecological malignancies

## Abstract

Gynecological malignancies are treated with a multi-disciplinary approach. There are two important factors, the stage and Karnofsky performance status scale (KPS) of patients, which guide the treatment strategy by single or multiple modalities in terms of surgery, radiotherapy, or chemotherapy. Various aspects are included in the workflow of gynecological malignancies, like screening, diagnosis in each individual, treatment modalities, and finally, follow-up to see for outcomes leading to the development of new research protocols. The quality data plays an important role in every step. Artificial Intelligence (AI) will play an important role if it is developed in the above-mentioned steps. AI is already established partially in every aspect of the management of gynecological cancer. It needs to be strengthened and incorporated further in a more robust form. This needs an association between clinicians, software engineers, and stakeholders. This article reviews the role of AI in various steps of the workflow of gynecological malignancies and discusses a few clinical aspects that may be researched to find solutions by AI.

## Introduction and background

Gynecological malignancies are around 19% of the cancer burden in the world [1]. Common gynecological malignancies are the cervix (6.5%), ovary (1.6%), and endometrium (4.5%). The management of these malignancies is a multi-disciplinary approach. Cancer cervix (Stage I-IVA) management includes primarily the role of surgery or radiotherapy and a lesser role of chemotherapy, limited to concurrent with radiotherapy. Cancer of the ovary is managed primarily with surgery and chemotherapy, and the radiotherapy role is almost negligible. Cancer of the endometrium is treated primarily by surgery and the moderate role of radiotherapy beyond early-stage disease.

AI is a type of digital computer system that parallels the way the human brain processes information. It is organized in a similar way that neurons in the brain are arranged, with their multiple neural nodes, and so are referred to as neural networks [2-3]. The rise of AI has led to the subsequent development of artificial neural networks (ANN), which consist of a dependable mathematical system that can interpret multifactorial data [4-7].

Clinical oncologists have their training by observing and assisting the true clinical cases in out-patients and in-patient departments. Regular decision-making on cancer cases is made through the knowledge and skills acquired by books, the internet, or mostly from teachers and peer colleagues. The decision-making in treatment may vary from institution to institution depending upon their resources of knowledge and training institutions.

**Figure 1:**
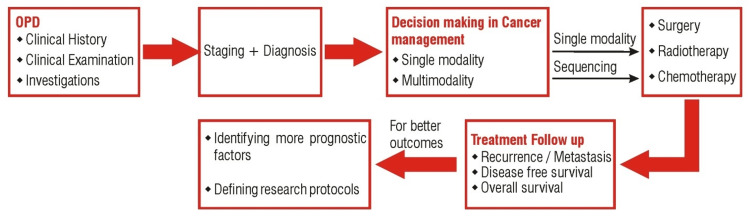
Workflow for the management of gynecological malignancies OPD: Outpatient Department

The incorporation of artificial intelligence (AI) in the management of cancer treatment may help to avoid bias in treatment strategies and may probably be more transparent, evidence-based, and better answerable in this medico-legal era. We would like to discuss the role of AI in different management strategies for gynecological malignancies from the perspective of a clinical oncologist. The workflow of management of gynecological cancers is demonstrated in Figure 1.

## Review

AI in screening and early detection of gynecological cancers

Screening guidelines are well established for cancer cervix worldwide. In recent years, AI has been revolutionizing cervical cancer screening and offering enhanced accuracy, efficiency, and potential for widespread impact. Cervical cancer is often linked to persistent human papillomavirus (HPV) infection and conventional screening methods like Pap smears have been pivotal. The limitations have prompted the search for more sophisticated solutions. Automated analysis of cytology samples is one of the examples. AI algorithms like convolutional neural network (CNN) and deep learning techniques can analyze Pap smears and liquid-based cytology samples with remarkable accuracy. They evaluate cell morphology, detecting subtle abnormalities that might be missed by human eyes [8].

Further, AI is enhancing its role in colposcopy and biopsy procedures. Colposcopy is the visual examination of the cervix and is crucial for diagnosis. AI assists clinicians by identifying suspicious areas, optimizing biopsy site selection, and minimizing false negatives. Cytology screening for high-grade cervical precancerous lesions is highly specific but less sensitive (50-70%) [9]. PAPNET, the first commercial automatic screening system [10], was used to rescreen the slides which were judged negatively by the cytologist. Another Thin prep imaging system, also a commercial screening product, could select the 22 most concerned fields of view (FOV) by proprietary algorithms, and if abnormal cells were found, the cytologist needed to manually screen the entire slide. These systems improved the sensitivity and efficiency, though these were not cost-effective and still some researchers continue to optimize the application of AI in cervical cytology. A typical automatic smear analysis system comprises of five stages image acquisition, preprocessing, segmentation, feature extraction, and classification. AI technology is applied in the segmentation and classification stages for the automatic analysis of the smear. Chankong et al. used the fuzzy C-means (FCM) clustering technique to segment a cervical single-cell image into the nucleus, cytoplasm, and background and obtained the morphological features to obtain automatic multi-label classification [11].

Ovarian cancer is often referred to as a silent killer challenge in women's health due to its late-stage diagnosis and high mortality rates. Over recent years, the fusion of AI with radiological science has introduced a transformative approach to ovarian cancer detection and prediction. AI-powered algorithms like CNN scrutinize radiological and ultrasound images with exceptional precision [12]. By identifying subtle abnormalities in ovarian masses, AI assists radiologists in early cancer detection. These systems utilize machine learning to predict the malignancy of ovarian masses based on diverse clinical and imaging data. These predictions guide clinicians in making informed decisions regarding treatment strategies. Erdemoglu et al. studied the role of AI in the prediction of intraepithelial neoplasia and endometrial cancer risks in pre- and post-menopausal women [13]. Various features related to history, symptoms, comorbid conditions, and thickness of endometrial samplings were collected. They concluded that age, body mass index, and endometrial thickness were important factors for predicting the risk (low, intermediate, and high risk) for endometrial cancer, in contrast to menopausal status and symptoms. We can interpret that AI aids in assessing patients' risk levels based on demographic, clinical, and histological data, which may help in the detection of gynecological cancer and early interventions can be planned.

AI in the treatment of gynecological cancers

Perspective of a Radiation Oncologist

Radiotherapy is a crucial treatment modality for cancer patients, aiming to target and eliminate cancer cells while minimizing damage to surrounding healthy tissues. The success of radiotherapy largely depends on accurate tumor targeting, dose planning, and minimizing radiation exposure to healthy tissues.

Radiotherapy treatment includes various planning and execution steps. The planning includes CT simulation, delineation (identification of tumor, delineation of regions where probability of microscopic spread is predicted, organs at risk which are to be saved from radiation injury), dose prescription, optimal planning, and plan evaluation by a clinical oncologist. The execution steps include the verification of position by images and quality assurance of treatment delivered.

Advanced techniques in radiotherapy are already utilizing AI in their planning and execution but it still consumes a lot of time. These may probably be solved with further advanced forms of AI, and verifications and various quality checks are included to evaluate and minimize errors. The role of AI can be seen and be further focused on various steps of radiotherapy planning and execution [14].

A. Treatment simulation-image acquisition, processing, and registration: The patient is immobilized to prevent motion and then medical images are acquired for radiotherapy planning. Presently, most of the patients are acquiring CT-based images and performing radiotherapy planning. A tumor is better delineated on MRI and if we utilize this modality to delineate structures, these MRI images need to be fused with CT images for planning purposes. The challenge in this scenario is that it is very difficult to acquire images in exactly the same position in both CT and MRI scans. A slight change in position of a few millimeters will have drastic errors and uncertainties in the delivery of optimum dose to the tumor and sparing of normal structures. AI has been working to generate synthetic CT images from MRI images of the brain and pelvis. There have been minimal dose differences observed between the treatment plans formulated using synthetic CT versus true CT images. The authors highlight the fact that this approach may have the dual benefit of decreasing the number of imaging appointments as well as decreasing the radiation exposures from CT scans [14,15].

In routine CT simulation planning also, AI can play a crucial role in ensuring the quality and accuracy of image acquisition. By detecting potential errors or inconsistencies in the acquired images, AI algorithms can flag issues that might otherwise go unnoticed. This feature enhances patient safety and reduces the risk of suboptimal treatments.

B. Delineation of structures: Accurate and consistent contouring is crucial in radiotherapy, as it directly impacts treatment efficacy and safety. In gynaecological cancers, the target volumes (which includes the gross tumor and regions of microscopic spread) and organs at risk (which are the rectum, urinary bladder, B/L femur, bone marrow, and small intestine) are typically performed manually by radiation oncologists, which can be time-consuming and influenced by subjectivity. AI-powered contouring solutions, driven by advanced machine learning algorithms, offer the potential to automate and optimize this process, addressing the limitations of manual contouring [16].

There are various AI Applications in contouring. First is atlas-based contouring, where AI can leverage pre-existing annotated atlases to enhance contouring accuracy and speed [17]. By using AI algorithms to deform and adapt existing contours to individual patient anatomy, clinicians can save time and ensure consistency in the delineation process. Second, multi-modality contouring, where AI can combine information from multiple imaging modalities (CT, PET, and/or MRI) to improve the precision of target volume delineation [18]. This multi-modality fusion enables a more comprehensive understanding of tumor boundaries, leading to improved treatment planning. Finally, knowledge-based contouring, where AI models can be trained using vast amounts of historical treatment data to create knowledge-based models [19]. These models offer intelligent suggestions for contouring based on similar cases, aiding radiation oncologists in generating high-quality treatment plans.

C. Dose prescription and optimal radiotherapy planning: AI can be utilized for predicting the optimal radiation dose distribution for individual gyne-oncology patients. By analyzing historical treatment data and patient-specific parameters, AI models can recommend personalized treatment plans that maximize tumor coverage while minimizing toxicity to healthy tissues. In cervix cancer, the delivery dose escalation may be seen in macroscopic disease (beyond FIGO stage I), where more doses need to be delivered. Further presence of pelvic lymph nodes may need a personalized treatment plan with more doses prescribed to it than routine. This needs to be planned in such a way that various organs at risk, like small bowel, bone marrow, femur heads, rectum, and bladder, are receiving doses within their tolerance limits, or else will lead to injury and morbidity of these structures. In a study by Kneepkens et al. on automated breast cancer plan generation, two AI model-based treatment plans (U-net model & cARF model) were evaluated against the corresponding manual plan. The U-net model resulted in a higher average and maximum dose to the planning target volume (PTV) and a slightly higher mean heart dose. The cARF model also had similar findings. In spite of these higher doses, 90-95% of the AI plans were considered clinically acceptable [20].

D. Plan evaluation: AI can aid in optimizing radiation dose distribution to achieve optimal tumor control while minimizing toxicity to surrounding healthy tissues. Knowledge-based models trained on historical treatment data can provide intelligent recommendations for personalized treatment plans. Clinical scenarios of cervix cancer being treated de novo with radiotherapy along with concurrent chemotherapy (case 1) or a case of post-operative radiotherapy (case 2) may have different planning objectives. Besides variation in doses to the macroscopic diseases in the de novo case (case 1) and microscopic dose in the post-operative case (case 2), there is a different thought process for bone marrow toxicity in both cases. In case 1, chemotherapy is adding to bone marrow toxicity and thus, evaluation doses for bone marrow will be more crucial in the first case scenario. Similarly, a patient with inflammatory bowel syndrome may like to receive a lesser dose to small bowel than any routine patient being planned for radiotherapy for cancer cervix or post-operative cancer endometrium.

E. Verification of treatment delivery: The success of radiotherapy treatment largely depends on accurate and reproducible patient positioning. Setup verification aims to verify that the patient is in the correct position for each treatment session. Cone beam CT (CBCT) is integrated with linear accelerators for image positioning verification. The quality of these CBCTs is lower than that of CT images on which treatment has been planned. AI has been used to improve the quality of CBCT images so that better position interpretation can be made, reducing set-up errors [21]. Various AI-powered solutions have emerged as promising tools to automate and optimize the setup verification process, offering improved precision and efficiency as compared to traditional methods.

Further, organ motion is also an important aspect contributing to change in target volumes to be treated. These can occur during treatment or on a daily basis. The rectum and bladder are two organs at risk in gynecological cancers where variable volumes are seen due to variable filling of feces and urine, respectively. Though various rectal and bladder protocols are followed in clinical practice but still perfect precision is compromised to some extent. AI can be used to study the variations and patient-specific dynamic motion management models may be developed to increase the precision in radiotherapy delivery.

Radiotherapy planning is a critical aspect of cancer treatment, as it directly impacts treatment efficacy and patient outcomes. The process involves delineating target volumes and organs at risk, optimizing radiation dose distribution, and ensuring patient-specific treatment plans. Manual planning can be time-consuming and subjective, leading to variations in treatment outcomes. AI-driven solutions offer the potential to streamline and optimize this process, enhancing treatment precision and personalization.

F. Quality assurance (QA) of the delivery: There are patient-specific and machine-specific QA assessments. In patient-specific assessments, AI tools are developed to check the accuracy of the treatment plan and treatment parameters. Further, they are also used for the accuracy between the planned dose and the delivered dose. In machine-specific QA, various AI algorithms are developed to predict errors for such as multi-leaf collimator positional errors. These AI models may help medical physicists accelerate their QA process and reduce the time for repeated tasks.

Perspective of a Gyne-oncologist

Surgery is an important aspect of the management of gynecological malignancies and has been performed for centuries. Though it is an effective part of treatment, complications can occur during gynecological surgery. Sometimes surgeries fail due to human errors, unknown reactions of the body, or inadequate equipment [22].

AI has been introduced recently in surgery, including gynecological surgery, with a strong root in imaging and navigation. Simultaneous processing of vast amounts of multimodal data, particularly imaging data, and incorporating diverse surgical videos with techniques focusing on feature detection and computer-assisted intervention will help in preoperative planning, intraoperative management, and post-operative recovery of gynecological cancers. AI is used to recognize patterns, classify images, or detect objects by analyzing digital images or videos through a process called computer vision [23]. These images would help to identify and predict any adverse outcomes during surgery and will assist the surgeon in clinical decision-making. For example, a case of cancer endometrium with previous pelvic surgery, in spite of CT and MRI, may have unforeseen adhesions due to previous surgeries. AI may help to tackle such problems on a real-time basis.

Surgery for gynaecological cancer planning and navigation has significantly improved through various radiological techniques (computed tomography, ultrasound, and magnetic resonance imaging). Minimal invasive surgery (MIS) and robot-assisted surgery (RAS) are novel advancements in gynaecological surgery. The introduction of AI in the robotic system will increase the capability of robots in perceiving the complex in-vivo environment and will help surgeons in decision making and performing the surgery with increased precision, safety, and efficiency. Preoperative molecular images like PET-CT, along with MRI images, are the starting point for many robotic procedures. Based on these images, the AI will help the surgeon to create a mental road map of the procedure. The surgeon may plan entry paths and trochar placements and in some cases, the AI may also guide the surgeon grossly to the area of target [24]. They result in decreased surgical trauma, less pain, shorter hospital stays, faster recovery, and fewer complications [25]. Robots are fatigue and tremor-resistant, have scalable motion, and have a greater range of axial movement which has been demonstrated to produce enhanced safety margins and accuracy of complex gynaecological surgeries. While the benefits of AI in laparoscopic surgery are considerable, there are also significant challenges and potential downsides to its integration. Lack of human judgement, lack of domain expertise, lack of data, bias in data, ethical issues, and cost are limitations in AI implementation [25].

A. Preoperative planning and intraoperative assistance: Areas in which AI has assisted gynecological surgery include those related to imaging and spatial awareness. Surgical intervention of the patient is preoperatively planned using the patient’s medical records and imaging. AI can aid the surgeon by providing better imaging before and during gynaecological surgery. The creation of three-dimensional printing (3DP) that replicates the surgical site is far superior to its two-dimensional (2D) counterpart, as it represents a more precise version of the actual model [26,27]. This fact is important in gynaecological malignancies present in the pelvic region where the injury to the bladder and ureter is a major concern for the surgeon and allows a more accurate preoperative plan, hence diminishing errors in the operating room [7].

Robotic simulators attached to an actual robot console can accurately represent what the surgeon will feel and experience in the operating room. Virtual reality training was compared to traditional apprenticeship training in a meta-analysis published in 2020, and the authors found that virtual reality training improved trainee efficiency, improved tissue handling, and reduced errors when compared to the traditional apprenticeship method of surgical training [28,29].

By combining simulator technology with 3D imaging, one can actually practice anatomically accurate surgeries of actual patients prior to doing surgery [29]. During surgeries, AI can display information or guidance during operation; it can monitor operations and send alerts. AI-based surgical systems can map out an approach to each patient’s surgical needs and guide and streamline surgical procedures.

There are four main areas of computer-aided intra-operative guidance in MIS that involve AI techniques: (1) shape instantiation, (2) endoscopic navigation, (3) tissue tracking, and (4) augmented reality (AR). Augmented reality consists of a computer that can reconstruct objects taken from the real world and enhance them virtually to create a more informative visual image [7,30,31].

These AR systems are essentially guidance systems that enable the surgeon to see the tumor of various gynaecological sites and its relationship to major intra-parenchymal vascular structures in real-time [29]. So it can help by increasing accuracy and decreasing operative time, so overall decreasing complication rate.

B. AI’s role in medical education and training in centers running post-graduate programmes on gynaecological malignancies: Overall, AI can provide learning tools for surgeons at all stages of their careers, tracking their performance or teaching them new skills. It also could help supplement the limited teaching capacity of specialized trained surgeons. Through AI, one can learn from a vast volume of data as it is not constrained by time or memory. In medical colleges where post-graduates are being trained to acquire knowledge and skills (including operative skills for gynaecological cancers), these AI tools will definitely supplement and accelerate the academic curriculum.

Although it may appear promising, there are several limitations of AI, including lack of data, bias in data, cost, and inability to handle uncertainty. As with any new technology, AI and each of its subfields are susceptible to unrealistic expectations from the media hype that can lead to significant disappointment and disillusionment is not a “magic bullet” that can yield answers to all questions [32]. There are also ethical concerns regarding the use of AI. Who would be responsible if AI-assisted surgery goes wrong; the programmer who created the software, the company that markets the software, the hospital that brought it or the physician who used it? Anyone who deploys AI models needs to make sure that the people using them understand their performance and their limitations. So at the end of the day, physicians will be accountable [16].

In conclusion, while AI's role in laparoscopic surgery is burgeoning and holds immense potential, it is not without its challenges. A balanced and thoughtful approach, considering both its advantages and disadvantages, is needed to harness AI's potential effectively and responsibly. As technology continues to advance, it is paramount that it is integrated in ways that prioritize patient care, uphold ethical standards, and enhance, rather than replace, the skills of our healthcare professionals.

Role of AI in chemotherapy

Traditional treatment approaches, such as chemotherapy and targeted therapy, have been the cornerstone of managing gynecological malignancies, especially ovarian cancer. However, the advent of artificial intelligence (AI) has introduced new avenues for optimizing treatment strategies, improving patient outcomes, and enhancing overall healthcare delivery [33].

Personalised Treatment Selection

AI-driven algorithms analyse vast data, including clinical records imaging, and genomic information, to develop personalized treatment plans. By integrating patient-specific factors, AI aids clinicians in selecting the most effective chemotherapy or targeted therapy regimen based on the tumor's molecular profile and the patient's overall health status. This approach reduces the risk of unnecessary treatments and minimizes potential side effects, ultimately enhancing the patient's quality of life [33].

Predictive Modelling

AI-powered predictive models can forecast patient response to specific chemotherapy agents or targeted therapies By analysing historical treatment outcomes and patient characteristics, these models help identify individuals who are more likely to benefit from a particular treatment, allowing clinicians to make informed decisions and adjust treatment plans accordingly. In a meta-analysis by Russo et al regarding AI predictive models of response to cytotoxic chemotherapy or targeted therapy for metastatic colorectal cancer patients, the results were promising in predicting the response to therapy and toxic side effects [34].

Treatment Monitoring

Continuous monitoring of patients undergoing chemotherapy and targeted therapy is crucial for evaluating treatment effectiveness and identifying potential adverse events. AI-powered tools analyze real-time patient data, enabling early detection of treatment-related complications and enabling timely interventions [35].

AI in research protocols following treatment outcomes

Cancer treatment is still evolving with advances in technology in imaging and treatment modalities (surgical or radiotherapy) along with newer chemotherapy and targeted therapies. Follow-up of cancer patients is very necessary to see the outcomes of treatment in terms of recurrences, metastasis, disease-free survivals and overall survivals. AI can be developed to play a very crucial role in analysing these outcomes that will be statistically helpful in various research protocols. This may help to develop various research protocols for treatment aiming to improve outcomes.

Demerits of AI

AI (Artificial Intelligence) has the potential to bring several advantages to the field of gynecological cancers. However, there are also some potential demerits to consider. The foremost demerit is that AI algorithms are dependent upon large amounts of high-quality data. If the data used is biased or flawed, it could lead to inaccurate or unreliable predictions. Secondly, though AI can assist in treatment planning and decision-making, it may lack the nuanced judgment and clinical experience of human experts.

Other important concerns will be the ethical issues and regulatory and legal challenges. The use of AI in clinical decision-making raises ethical considerations, such as the potential for delegating critical decisions to machines and the responsibility for errors or adverse outcomes [36]. Further, AI in medical practice is subject to regulatory approvals and legal considerations. Ensuring compliance with standards and regulations will be a crucial issue [37]. Some other issues like patient privacy and security, finances involved, and technical expertise will always be challenges with AI implementation.

## Conclusions

AI will definitely promise to transform practices of gynecological cancers in different treatment modalities of surgery, radiation therapy, and chemotherapy, along with practices in screening and diagnostics. The fear of failing in any aspect will always be there in terms of software nuisances and misuse by operators. These fears need to be governed by some competent authorities and strict guidelines to be issued by regulatory bodies so that the best use of AI can be made.
